# Inglämnlagare - a tool for restructuring Swedish site data for statistical analysis

**DOI:** 10.12688/f1000research.126484.1

**Published:** 2022-11-23

**Authors:** Daniel Löwenborg, Filipp Antomonov

**Affiliations:** 1 Archaeology and Ancient History, Uppsala University, Uppsala, 751 26, Sweden

**Keywords:** GIS, Landscape Analysis, Sweden, Historic Environment Record, data restructuring, python, regular expressions

## Abstract

***Background:*** This paper presents a new software tool, *Inglämnlagare,* developed to be open-source, that restructures information about ancient remains in Sweden for analysis. The background is a new version of the ancient sites database, the Historic Environment Record, curated by the Swedish National Heritage Board, that was launched in 2018 with a new database model that structures the information differently compared to previous versions.

**Methods**: The program, written in Python programming language, has multicore support in order to improve performance for large files and uses regular expressions to extract information about individual features of composite sites. Such features, together with their summed amount, are written as new individual fields to a comma-separated value file. The program is delivered as a source script file that can be executed in any Python environment.

**Use cases**: As an example of use, a case study of exploring graves of rectangular shape found within Sweden is provided. The use case also describes the different steps involved in preparing the data in QGIS to run the program, as well as some methods to efficiently analyse and visualize the output.

**Conclusions:** *Inglämnlagare* will make more information from the Swedish record of ancient sites accessible for research and can be used to explore different content of the record more efficiently than previously possible. While the tool is written specifically for this dataset it also provides an example of how open-source tools can be used for data wrangling making information designed for a specific purpose, such as online dissemination, appropriate for analysis.

## Introduction

Many fields of archaeological research are revitalised as digital technology enables the use of large volumes of data for analysis ( Huggett 2020). Increased access to information from the internet and online databases enables both completely new approaches to interpret prehistory, and also makes it possible to revisit previous results with new computational methodologies to verify or challenge previous theories ( Cooper & Green 2016, Gillings, Hacıgüzeller & Lock 2022). However, access to data is not enough, and it is necessary that data structures are appropriate for the analytical tools used. Especially reusing data from external sources, that may have been collected for other purposes, often means that the data will need more or less extensive preparation before analysis ( Gupta 2020). Sweden has good access to geodata about archaeological sites since the Swedish National Heritage Board (NHB, Swedish: *Riksantikvarieämbetet, RAÄ* ) has provided a digital record of sites as open access since 2006 when the Archaeological Sites and Monuments Information System (FMIS) were launched ( Larsson & Löwenborg 2020). In 2018 this was replaced by the updated new Historic Environment Record (HER, Swedish: *Kulturmiljöregistret KMR*), which in addition to protected sites also has information about archaeological investigations, reports and to some extent also documentation ( Larsson, Smith, Sohlenius & Klafver 2017). HER currently holds about 800 000 sites, with different levels of legal protection. Since the purpose of this database was to record the antiquarian status of each site, the data structure was not primarily designed to facilitate analysis ( Löwenborg 2007, Sohlenius 2014). However, the information in the record still holds much data that will be of interest for research purposes, to understand regional differences through spatial analysis and approaches from landscape archaeology.

One aspect that makes it more difficult to work with the data is that several sites are composites that contain information about individual features that are part of a larger site. Typically, a burial ground has information about how many graves have been observed during the survey and the classification of their external features. This is described as “grave type”, “shape” and “construction details”, as defined in the *Lämningstyplista* . Some graves have two variables for construction, to describe different aspects, and there are also a few occurrences of a variable type “ *fyllning*” that only has one value: “ *Rödockrafärgad sand*” *i.e.* a grave that contains red ochre sand. Thus, to describe a grave there can be up to four values plus the number of graves of each type. The most common grave type, the mode value, is “stone settings” ( *stensättning*) with shape “round” ( *rund*) and construction “earth filled” ( *övertorvad*), as seen in Table 1 below. These represent almost 50% of all graves in HER. In this paper, we will present a new open source python program that uses regular expressions to restructure Swedish HER data to a format that is more suitable for statistical analysis. While the program is specifically designed for this particular dataset, the principles for restructuring data would be relevant in other cases where a compact data structure, suitable for easy dissemination of data online, needs to be reorganised for analysis. This will be illustrated with an example of extracting all the graves in Sweden and looking at some patterns in this material. We will also present a use case of how to prepare the data to facilitate analysis.

**Table 1.** The top 10 most common categories of grave types from HER, after extraction with the *Inglämnlagare *program.

Grave type	Number of graves
Stensättning – Konstruktion: Övertorvad; Form: Rund	251,673
Stensättning – Konstruktion: Stenfylld; Form: Rund	70,077
Hög – Form: Rund	64,468
Röse – Form: Rund	18,935
Grav markerad av sten/block – Typ: Rest sten	18,388
Flatmarksgrav	7,128
Hög	7,085
Grav - uppgift om typ saknas	5,843
Stensättning – Konstruktion: Övertorvad; Form: Rektangulär	5,447
Stensättning	4,608

The former FMIS system had a different solution for how information about grave features were included in the data compared to the new HER record. In FMIS, information about individual features (graves) that are part of a composite site (burial ground) was in a separate table, called “nil”, that was included with the exported shapefiles. The nil table had an identification key ( *samsatt_id*) that related to the geometries in the shapefile so that information about the individual graves could be related to the sites. The information about features was divided into separate rows and fields, which together provided information about the number of different graves at a burial ground. Since the information was split up into different fields and rows, it was necessary to rebuild the data in the record in order to perform statistical analysis. This could be done in different ways with different software tools ( Löwenborg 2007, Ma 2020). With the transition to the HER system, a different solution for presenting the data was used, which make use of the new data format for geodata; GeoPackage (GPKG) developed by the Open Geospatial Consortium. One of the benefits of the GPKG format is the possibility to include long string values in the attribute table. Rather than keeping the information about individual features in a separate table, and in multiple rows and fields, the HER format makes an array of all this information, and includes that in a field in the GPKG dataset. Information about the transition from FMIS to HER, the quality work involved in this, and some information about how data was restructured are described on the NHB website. The new variable with information about features at a site is called “ *ing_lamn*” (Swe: *ingående lämningar*) and would look like this for the mode grave type described above:

{"lamningstyp":"Stensättning","antal":X,"egenskap":"Konstruktion:Övertorvad, Form: Rund"}


‘X’ here is the number of this particular type of graves at a site. Since a burial ground often has graves of different types and kinds, each type will be represented by a similar string, and burial grounds could thus have a string of considerable length to describe all the types of graves there. An example from HER, L2014:3046, with one of the larger and more complex sites with many different grave types looks like this:

[{"lamningstyp":"Boplats","antal":1,"egenskap":null},{"lamningstyp":"Grav - uppgift om typ saknas","antal":3,"egenskap":null},{"lamningstyp":"Grav markerad av sten/block","antal":300,"egenskap":"Typ:Rest sten"},{"lamningstyp":"Stenkrets/stenrad","antal":1,"egenskap":"Form:Övrig"},{"lamningstyp":"Stensättning","antal":1,"egenskap":"Konstruktion:Övertorvad, Form:Skeppsformig"},{"lamningstyp":"Stensättning","antal":254,"egenskap":"Konstruktion:Övertorvad, Form:Rund"},{"lamningstyp":"Stensättning","antal":30,"egenskap":"Konstruktion:Stenfylld, Form:Rund"},{"lamningstyp":"Röse","antal":1,"egenskap":"Form:Rund"},{"lamningstyp":"Hög","antal":1,"egenskap":"Form:Rund"},{"lamningstyp":"Stensättning","antal":6,"egenskap":"Konstruktion:Övertorvad, Form:Kvadratisk"},{"lamningstyp":"Stensättning","antal":4,"egenskap":"Konstruktion:Övertorvad, Form:Rektangulär"},{"lamningstyp":"Stensättning","antal":8,"egenskap":"Konstruktion:Övertorvad, Form:Triangulär"},{"lamningstyp":"Stenkrets/stenrad","antal":15,"egenskap":"Konstruktion:Övrig, Form:Domarring"},{"lamningstyp":"Stenkrets/stenrad","antal":34,"egenskap":"Form:Kvadratisk"},{"lamningstyp":"Stenkrets/stenrad","antal":4,"egenskap":"Form:Rektangulär"}]


For graves registered as individual, single graves, the structure is different. These would generally be solitary graves, or in sparse groups, where each grave is recorded as a unique object in the register. These are usually a point but could also have a polygon geometry. For these objects, the information about grave type is in the variable “ *lamningtyp*” ( *i.e. stensättning*) and information about shape and construction details of these, if any, are recorded in the variable “ *egenskap*” ( *i.e. Form: Rund, Konstruktion: Stenfylld*). If no features are used to describe the grave, the “ *egenskap*” variable will be ‘NULL’. There is no number associated with these objects since they all represent one single grave.

Having all information about burial types collected in one file is a great benefit compared to the previous version with the “nil” layer. The nature of the information where the data is split up into different variables for individual graves, and the complex structure of the “ *ing_lamn*” string, still means that it cannot be analysed statistically without prior remodelling. A fairly simple question as to how many burials there are at a burial ground site means that all the “ *antal*” (number) values would need to be summed up for each site. More complex queries, such as how many stone settings, mounds or other types of graves there are, mean that the “ *antal*” needs to be summed separately. To also account for the different shapes and construction types makes it quite complex to extract the information for anything more than a few individual sites. In order to calculate this for regions, or even at a national level, it will first be necessary to restructure the data completely. The program *Inglämnlagare*, written by Filipp Antomonov, does this by extracting all different categories of burial types and adding the number of these into separate fields for each type.

## Methods

In order for *Inglämnlagare* ( Löwenborg 2022b) to work correctly, the data has to be supplied in a comma-separated value file and include a *fid* attribute to each object in the database, needed for sorting the data. The program, provided as a single script file *Ing.py,* is written in Python programming language (RRID:SCR_008394) and has multicore support in order to improve processing performance of large files. The script can be run in any Python environment (including directly in QGIS) or compiled as a standalone executable for Linux, Windows or Macintosh operating systems to be used without an installed Python environment. A general outline of the process of extracting information from HER data using *Inglämnlagare* is described in the use case below.

### Use case

To get the most out of the program *Inglämnlagare* there are several steps needed both before and after, to ensure reliable results that are suited for further analysis. Here we will present a short use case of the steps involved, working with all graves from HER *Lämningar*, and extracting a subset for analysis. For preparation of data beforehand, and for analysing the results, we will use the Open-Source GIS (Geographical Information System) program QGIS (RRID:SCR_018507) version 3.22.11. The HER *Lämningar* is available as open data from the Swedish NHB at their open data portal. To have all the data, it is the GPKG version that should be used, since the Shapefile version is missing the *ing_lamn* variable. In the version that was used here, downloaded on August 16, 2022, there are 799,272 objects available, divided into 507,188 point features, 77,242 lines and 214,842 polygons. For regional analysis where the individual geometries are not needed, it is recommended to first convert all features to points (centroids) and merge them to one layer so that all analysis can be done on the same dataset. Converting to points might create duplicates from multipart features, which should be removed before analysis.

Depending on the questions at hand, it will usually be relevant to make a selection of the data in order to limit the number of objects before continuing. In this example we are only interested in actual archaeological sites. The register holds other categories too in the variable “ *antikv_bed*”, describing the legal status of each site. In this case, we want *‘Fornlämning’* (ancient remains) and ‘ *Ingen antikvarisk bedömning*’ that includes sites that have been excavated and removed, thus losing their legal protection as ancient sites. We might want to include ‘ *Möjlig fornlämning*’ (possible ancient site) but the remaining categories would not be relevant in this case (see the NHB website for information about the classification of sites).

The key variable for selecting relevant sites is “ *lamningtyp*” as this holds information about the general category of each site. Full descriptions of these categories are in *Lämningstyplistan*, available from NHB. This list holds both the formal definition of each category, and also explains how graves can be either as simple categories, where each object is one type of grave, or be part of composite sites, where graves can be included in either burial grounds ( *Gravfält*) or burial and settlement sites ( *Grav- och boplatsområde*). For this example, graves that would not be prehistoric were excluded. The following query was used in QGIS to select all categories of sites in the HER database that would contain graves according to the criteria above:

"lamningtyp" = 'Grav - uppgift om typ saknas' OR "lamningtyp" = 'Grav markerad av sten/block' OR "lamningtyp" = 'Grav övrig' OR "lamningtyp" = 'Gravklot' OR "lamningtyp" = 'Grav- och boplatsområde' OR "lamningtyp" = 'Gravfält' OR "lamningtyp" = 'Hög' OR "lamningtyp" = 'Järnåldersdös' OR "lamningtyp" = 'Stenkammargrav' OR "lamningtyp" = 'Stenkistgrav' OR "lamningtyp" = 'Röse' OR "lamningtyp" = 'Stenkrets/stenrad' OR "lamningtyp" = 'Stensättning'


This query returned 127,641 objects. Before proceeding, it might be practical to delete fields with data not needed later, such as antiquarian and meta information about data creation. If working with a large selection of sites, it might also be advisable to write XY coordinates to each site in the attribute table. This can simplify the process of bringing the data back into GIS software after analysis, by adding points based on coordinates. Another option would be to join the output of the analysis to the original point layer, but that can be difficult with large volumes of data. Following this, the data can be exported to a CSV file and parsed by the program.

As an example, below, *Inglämnlagare* is executed in QGIS under Windows, via the built-in Python console, located in the *Plugins* menu. Start by defining the variable *ing,* by entering the path to the script file Ing.py:

ing = r"C:\GIS\Ing.py"


**Figure 1.**
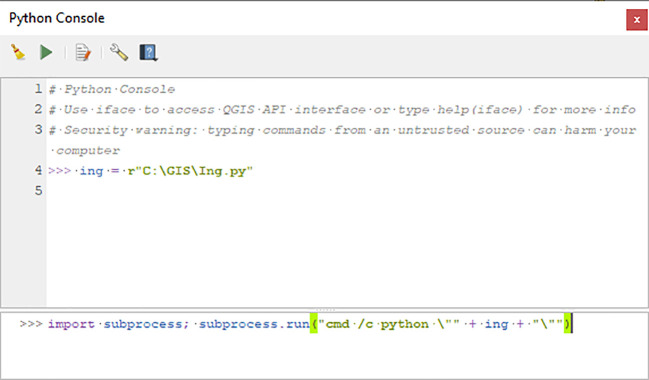
The two lines added in the QGIS Python console to run *Inglämnlagare* under Windows.

After replacing the path above with a correct one for the script’s location on the machine, the process is then initiated by entering the following string in the console, as illustrated in Figure 1:

import subprocess; subprocess.run("cmd /c python \"" + ing + "\"")


However, if the machine, regardless of the operating system, already has an installed Python environment, *Inglämnlagare* can simply be started in the shell via the command *python Ing.py.* And in the case of a compiled executable, just by running it. The program begins by asking for an existing input file and a desired output file. If only a filename is entered for the input, the location is assumed to be the one of the program file; in case of the same for the output – that of the input file. A list of all possible combinations of site types and attributes is then created. First, all the combinations of *lamningtyp* and *egenskap* are collected. After this, the list continues to be expanded with the information in the variable *ing_lamn* so that all unique combinations of objects are found. To identify relevant segments of information within the strings, regular expressions are used throughout the program. Regular expressions are a set of powerful functions within computational linguistics for working with patterns in text, to manipulate or extract information. For example, the command *\d+* finds one or more numerical values – from the string below, the value “6” would be returned:

*{"lamningstyp":"Stensättning","antal":6,"egenskap":"Konstruktion:Övertorvad, Form:Rund"}* 


The same principle is used to identify grave type, shape and construction detail, using the structure of the “ *ing_lamn*” variable. When all possible types of graves have been identified, these are added as new fields, so that the example above would be a new variable called: “ *Stensättning – Konstruktion: Övertorvad; Form: Rund*”. For each row in the file, the number of features (“ *antal*”) is then extracted and written as a value for the object, identified by the fid. For the single graves where no number is provided, the value is set to “1”.

## Results

The output of *Inglämnlagare* is written to a new CSV, with all the input values intact, and with the addition of fields for all possible combinations of values. This can be a very long list, depending on what selection of sites was used as input, and the complexity of these in terms of possible values. Extracting all the graves contained in the HER database gives a total of 507,078 individual graves from 127 641 sites. The graves are classified into 135 different grave types. Stone settings are both the most common type of grave, and are also represented by the most variables for different combinations, with 53 different types in total.

Many graves are of the same type. The top five categories hold 83,5 % of all the graves, and the most common types are presented in Table 1. At the other end of the scale, there are 14 types of graves that are represented by only one object each, such as one: *Stensättning – Konstruktion: Övertorvad; Fyllning: Rödockrafärgad sand; Form: Oval.* Some of the less frequent types of graves might be interesting to explore closer as they could represent specific burial practices that can inform us of cultural and social prehistoric phenomena.

For further spatial analysis the data can be imported back into QGIS, and for an efficient workflow there are a few suggested steps to prepare the data still. Including more general composite categories, such as ' *Grav- och boplatsområde*' as in the example query provided above, means that when all objects in “ *ing_lamn*” will be written as a new variable in a field, the number of fields might become very large, and will probably contain information that is not needed. In this case, the resulting table holds 356 fields, where a large number are features from settlements, such as hearths, post-holes, houses etc. Deleting unwanted fields from a table might be difficult, especially in GIS software, so a faster approach might be to export the result to a new layer, and only include the desired fields in the export. This can be achieved during the setup of the export to a new GPKG, by excluding all fields except those that should be kept for further analysis, making the data more manageable. When all fields that contained non-grave information had been removed, only the 135 variables with fields containing graves remain. As this still is a fairly large number of variables, that might contain more information than needed for the analysis, a useful technique would be to combine the categories of interest into a new field. For example, we might only be interested in graves of a particular shape, but not all the different descriptions of construction of these.

One example of grave type that might be out of interest for further analysis are rectangular stone settings, which often have been associated with the early phases of Christianity at the end of the Viking period ( Therus 2019, 42). If we would single out stone settings of rectangular shape, we would still be left with five categories, depending on the construction types. It might further be of benefit to combine these with other grave types, such as rectangular graves constructed with erected stones. These antiquarian classifications may or may not be relevant to the archaeological questions we have, so it is up to the research questions to define analytical units. Assuming we would like to explore rectangular graves, it might be relevant to include both kinds of rectangular graves for a total of 8,004 individual graves, distributed on 3,578 different sites, listed in Table 2.

**Table 2.** The nine different categories of rectangular graves that exist in the database, except rectangular mounds and cairns that were left out. The differences in the construction types are “boulder stones”, “erected stones”, “supported stones”, “no fill”, “stone filled”, “earth-filled”, “other” or without value.

Grave type	Number of graves
Stenkrets/stenrad – Form: Rektangulär	146
Stenkrets/stenrad – Konstruktion: Klumpstenar; Form: Rektangulär	6
Stenkrets/stenrad – Konstruktion: Resta stenar; Form: Rektangulär	8
Stenkrets/stenrad – Konstruktion: Uppallade stenar; Form: Rektangulär	5
Stensättning – Form: Rektangulär	156
Stensättning – Konstruktion: Ofylld; Form: Rektangulär	142
Stensättning – Konstruktion: Stenfylld; Form: Rektangulär	1,718
Stensättning – Konstruktion: Övertorvad; Form: Rektangulär	5,447
Stensättning – Konstruktion: Övrig; Form: Rektangulär	376

**Figure 2.**
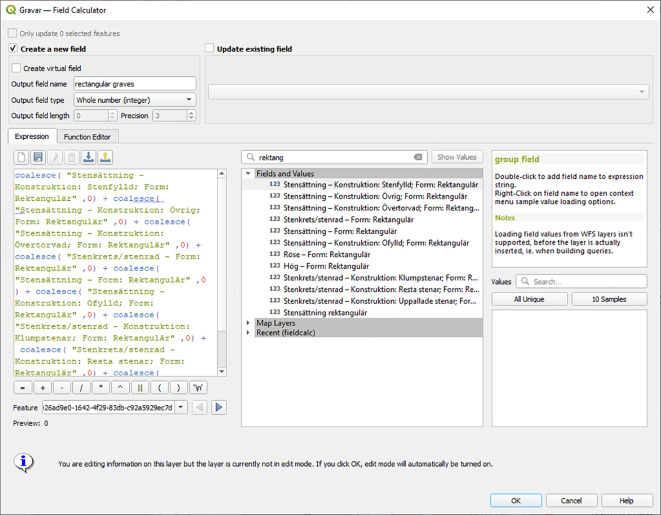
The Field Calculator in QGIS, where the partial string ‘rektang’ is used to filter for all fields with ‘rektangulär’ in the field name. This simplifies the identification of fields that should be included for extracting the values to the new field “rectangular graves”. Note that rectangular mounds and cairns were not included here, as they are more likely to represent a different cultural expression. Screenshot from the program QGIS version 3.22.11.

Unless analysis will be made of all the characteristics of the nine different fields containing information about rectangular stone settings and rectangular graves of erected stones, further analysis would be more efficient if all the rectangular graves are added up to a single new field, that has the sum of all graves of this type. A useful function for doing this is the possibility to filter variables by parts of field names in the QGIS Field Calculator as illustrated in the central section of Figure 2.

Depending on what software is used for further analysis, it is also important to be aware of how NULL values are handled. Adding up fields that include any NULL in QGIS values would give a NULL output for the total. To avoid this, NULL values could be changed to ‘0’, or as in the example in Figure 2, the SQL expression COALESCE could be used, in order to regard NULL values as 0 for the calculation and add up any real numbers. Summed values such as the new field “Rectangular graves” would make further aggregation into areal units fast and efficient.

**Figure 3.**
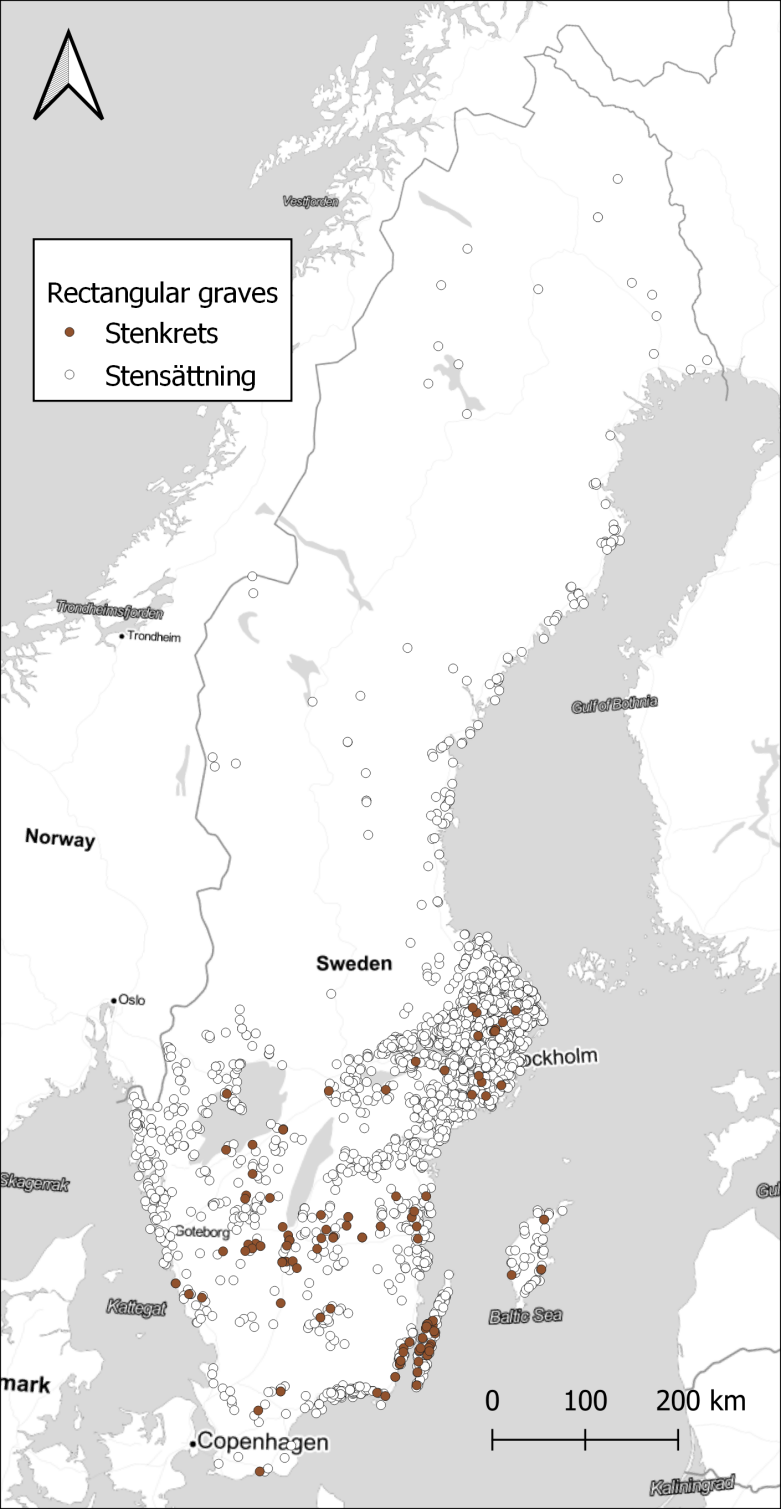
Distribution of sites with 1 or more rectangular graves, either stone settings or erected stones. Data from NHB/RAÄ Kulturmiljöregister (KMR), 2022-08-16. Map tiles by Stamen Design, under CC BY 3.0. Data by OpenStreetMap, under ODbL.

For a small number of sites, it might well be enough to plot them on a map to see the distribution, as in Figure 3. The version of rectangular graves that have been classified as erected stones are only present in the southern part of Sweden, with a few concentrations for example around the island of *Öland.*

**Figure 4.**
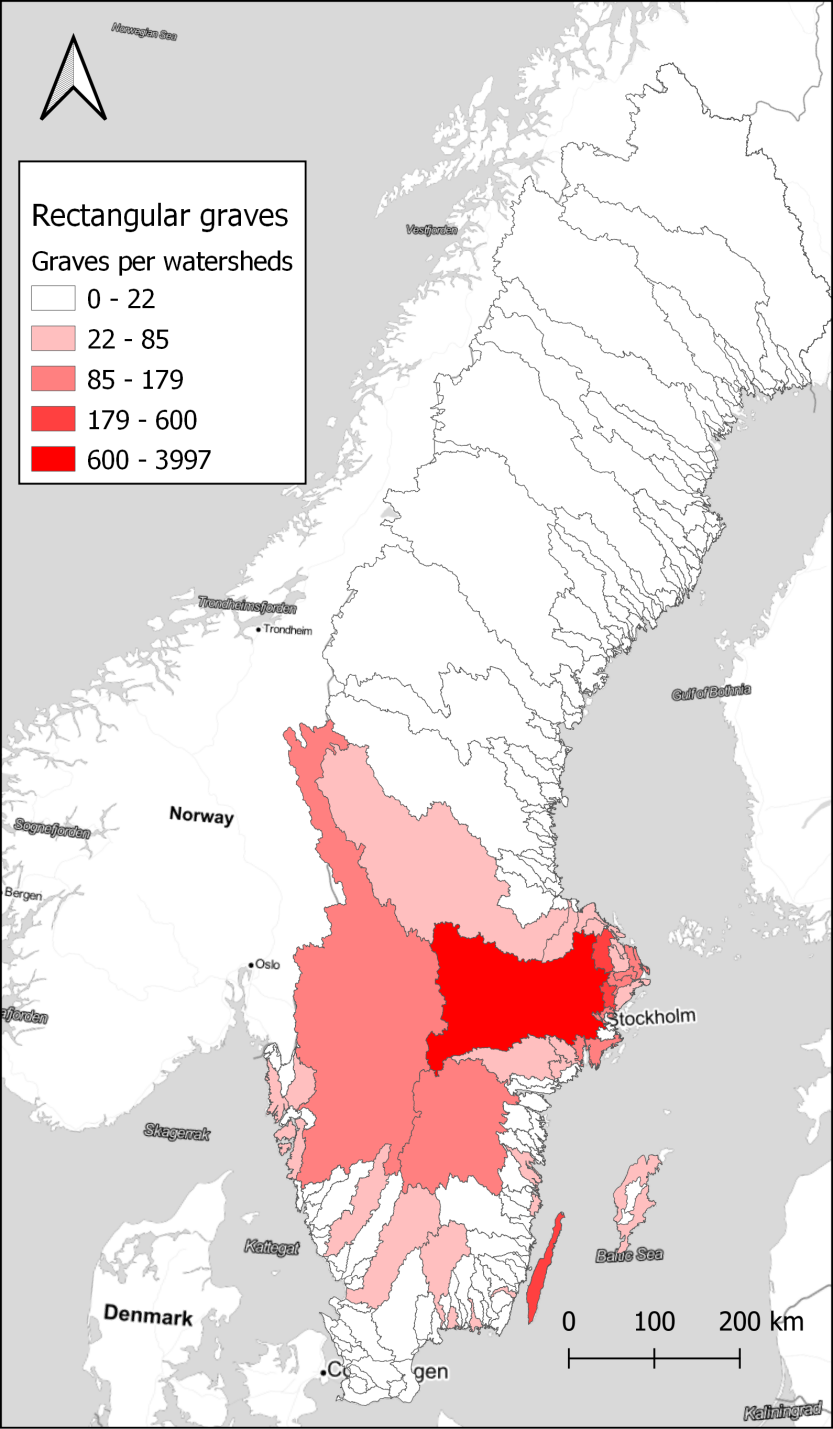
The total number of rectangular graves within watersheds. Data from NHB/RAÄ Kulturmiljöregister (KMR), 2022-08-16 and SMHI, the Swedish Meteorological and Hydrological Institute, Huvudavrinningsområden 2016. Map tiles by Stamen Design, under CC BY 3.0. Data by OpenStreetMap, under ODbL.

**Figure 5.**
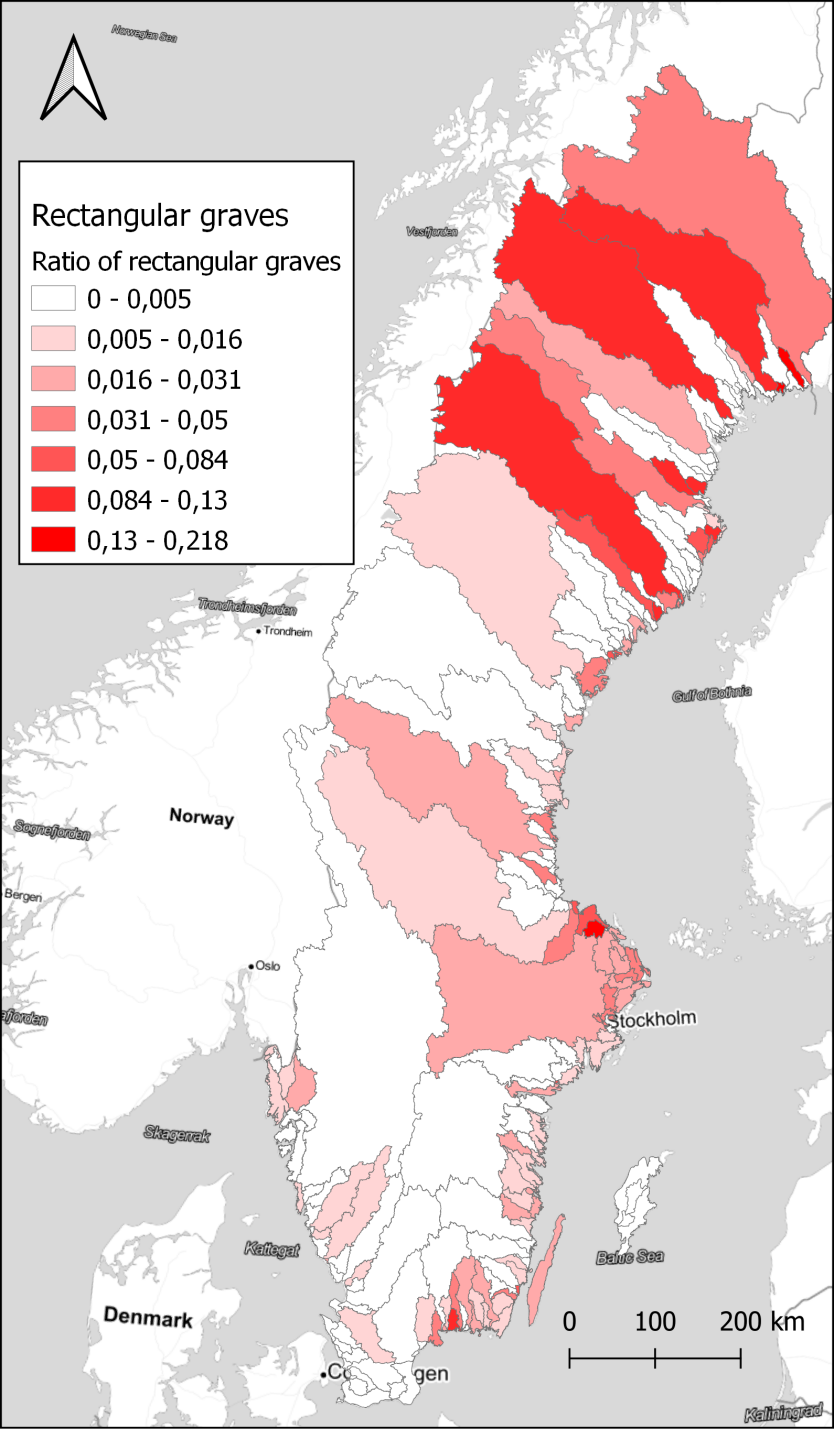
The ratio of rectangular graves compared to the total number of graves within each watershed. Data from NHB/RAÄ Kulturmiljöregister (KMR), 2022-08-16 and SMHI, the Swedish Meteorological and Hydrological Institute, Huvudavrinningsområden 2016. Map tiles by Stamen Design, under CC BY 3.0. Data by OpenStreetMap, under ODbL.

With a larger number of sites, it is better to aggregate the results to visualise spatial differences. Here, the choice was made to use watersheds as the unit of spatial aggregation. Watersheds have been suggested as a geographical unit that might have relevance in this area during the Late Iron Age, as a possible natural background to the early medieval hundred district organisation ( Löwenborg 2008). Looking at how the rectangular graves are distributed on the watersheds, there is a concentration in the central parts around Lake *Mälaren* and on *Öland*, as seen in Figure 4. However, looking at the ratio of rectangular graves compared to the total number of graves within each watershed displays a different pattern, in Figure 5. A few small areas on the coast in Uppland and Blekinge show high concentrations, and also some areas in the far north. Comparing with Figure 3, we can assume that rectangular graves primarily are a coastal phenomenon also in the north, with a few exceptions in the inland. While the substantial differences in the size of the watersheds might be obscuring some relevant patterns, it illustrates the potential in exploring the data in different ways. With the large amount of data that restructuring the original dataset of sites provides, there are great opportunities to explore different patterns that otherwise would be difficult to observe.

## Discussion

The restructuring of data that *Inglämnlagare* provides makes the full range of information of the HER variable “ *ing_lamn*” available for analysis. While the information in the array variable can be queried and extracted for limited questions already as it is, the possibility to work with quantitative methods on the whole dataset opens new ways of approaching the material. There is much specific information that might be useful here, such as the characterisation of features that are part of settlements, and thus not visible from the site level information by itself. As the origin of the record is the systematics surveys that were carried out in Sweden during the 20th century, sites that have been recorded are primarily those that are visible above ground ( Jensen 1997). With half a million graves in the database, this material can lend itself to intra-regional analysis, something that was an influential branch of Swedish archaeology, especially in the 1960s and 1970s ( Ambrosiani 1964, Hyenstrand 1974). It is, however, important to remember that there are substantial quality issues with the record. The information was collected over a long time, and policies and guidelines changed considerably during this period. In some parts of Sweden there has been specific rounds of survey in later time, such as within the project *Skog & Historia* ( Hermodsson 2003). This means that some areas have been much more thoroughly surveyed compared to others, and different definitions of what should be recorded mean that there are differences in which sites were recorded in different areas, depending on at what point in time they were surveyed ( Jensen 2006). The record is also incomplete since much of the archaeological remains are not visible above ground. When archaeological investigations are carried out there are often new sites found, including graves and burial grounds ( Löwenborg 2010). Any analysis of the record must thus take into consideration these limitations.

The HER record is now updated with the results of excavations at a general level, with information about the features found during excavations, such as graves, hearths, houses etc. However, the full documentation from excavations is still only partially collected, with find lists as a start, but the full documentation with GIS data and other databases is currently not made available in a systematic way. With the ongoing development of a digital archive at the NHB, and the newly founded national infrastructure for digital archaeology in Sweden, SweDigArch, this will probably change in the future. More data will thus be collected and made available for Swedish archaeology in the future. With rich data from excavations, both the volume and complexity of data will increase considerably, and there will be a need for new tools to make the most of this information, to lay out the ever-growing puzzle of information about the past.

## Data Availability

The original data used is available as Open Data from the Swedish National Heritage Board from https://pub.raa.se/ (accessed on August 16, 2022). Zenodo: KMR_Gravar_20220816. https://doi.org/10.5281/zenodo.7229877 ( Löwenborg 2022a). This project contains the following underlying data:
-
KMR_Gravar_20220816.gpkg (dataset of graves for all of Sweden, as a subset of the HER data above, and restructured with the Inglämnlagare program).

## Software availability

The source code for the program *Inglämnlagare* is available from: https://github.com/lowenborg/Inglamnlagare

Archived source code available from: https://doi.org/10.5281/zenodo.7230060 ( Löwenborg. 2022b).

License: GPL-2.0 license.
