# Supplementary material for: Inglämnlagare: a tool for restructuring Swedish HER record site data for statistical analysis
Source: F1000Res. 2023 Oct 17;11:1370. Originally published 2022 Nov 23. [Version 2] doi: 10.12688/f1000research.126484.2 (PMC10660290; doi:10.12688/f1000research.126484.2)
Supplement: Peer review history

# Reviewer report: Reviewer response for version 2

**DOI:** 10.5256/f1000research.157510.r222117

**Published:** 2023-11-27

**Authors:** Joe Roe

**Affiliations:** 1 Institut Für Archäologische Wissenschaften, Universität Bern, Bern, Switzerland

The article describes a Python script that transforms data from the Swedish Historic Environment Record (HER) into a format more amenable for programmatic data analysis. *Inglämnlagare* addresses an increasingly common problem in computational archaeology: making institutional open data resources, which are often designed with specific use-cases and/or end-user software in mind, available for general use in modern data analysis pipelines. In that sense reminds me of a similar recently-developed R tool for processing sites records from US federal agencies ^1^.

 The use of the tool is clearly illustrated through a step-by-step case study of early Christian rectangular graves. It presumes an existing basic familiarity with SQL and the use of delimited text formats in a GIS environment. Users of the software will find this section especially useful because the documentation that accompanies the software itself (as of writing) is quite sparse, and mostly written in Swedish. Incidentally, the article also serves as a useful introduction to the structure of the Swedish HER in English, which helps make it more accessible to international research.

 The tool itself is technically straightforward, consisting of a set of Python functions that parse HER data in CSV format, extract specified key–value pairs, and adds columns summarising the frequency of cooccurrence of each to the input data. Specifically, this function is applied to the "ing_lamn" field ( *ingående lämningar*), which encodes arbitrary additional properties of the site as nested key–value pairs in what appears to  be JSON format. The user must convert the GPKG provided by the HER data portal to CSV format beforehand, and additional pre-processing is recommended in some circumstances. These are probably easy enough tasks for most potential users, but incorporating them into the software in a future version could be a way to make the software accessible to a broader audience. A command line interface facilitates the use of the tool outside of a Python environment (e.g. in QGIS, as described here, but also in scripted analyses or in other environments such as R) and includes multicore support to speed up the processing of larger datasets.

 The implementation of this functionality relies heavily on regular expressions (somewhat misleadingly described here as something "within [?] computational linguistics"; in actuality a widely-used string pattern matching tool that has no model of natural language structures). I find this choice a little surprising, because the data in the  appears to be JSON and an efficient JSON parser is built into the Python standard library. Conversely, JSON is not a regular language so it is provably impossible to reliably parse it with regular expressions ^2^. It could well be that regular expressions were a compromise for performance reasons, or that I'm mistaken in my assumption that the contents of the "ing_lamn" is valid JSON. But either way, a concrete result of this choice is that that parsing is fragile and will almost certainly fail if the HER ever changes its output format for the ing_lamn field. This is always a problem with tools relient on external data structures or APIs, but is something users of *Inglämnlagare* should bear in mind if they want to ensure that their use of it is reproducible in the future.

 As other reviewers have noted, the potential use cases of the tool are realistically limited to the specific task of 'rectangling' site property data from the Swedish HER. Users seeking to obtain a similar result with data from other sources will likely find it easier to use general tools for 'data wrangling', such as *tidyr* and related R packages ^3^, the Python package *pandas* ^4^, or *OpenRefine* , rather than trying to adapt this script. Nevertheless, writing software the does one thing and does it well is nothing to criticise! *Inglämnlagare* is a robust addition to the computational archaeologist's toolkit and will likely prove useful both to researchers that already work with Swedish HER and those who want to incorporate into broader analyses or reconcile it with other datasets.

Are the conclusions about the tool and its performance adequately supported by the findings presented in the article?

Yes

Is the rationale for developing the new software tool clearly explained?

Yes

Is the description of the software tool technically sound?

Yes

Are sufficient details of the code, methods and analysis (if applicable) provided to allow replication of the software development and its use by others?

Yes

Is sufficient information provided to allow interpretation of the expected output datasets and any results generated using the tool?

Yes

Reviewer Expertise:

Digital archaeology, computational archaeology, research software engineering

I confirm that I have read this submission and believe that I have an appropriate level of expertise to confirm that it is of an acceptable scientific standard.

## References

[1] : ArchaeoSRP. *Advances in Archaeological Practice* .2023; 10.1017/aap.2023.22 1-11 10.1017/aap.2023.22

[2] Is JSON a Regular Language?. *Stack Exchange* . Reference source

[3] : tidyr: Tidy Messy Data. R package version 1.3.0.2023; Reference source

[4] pandas-dev/pandas: Pandas (v2.1.3). *Zenodo* .2023; 10.5281/zenodo.10107975

# Reviewer report: Reviewer response for version 2

**DOI:** 10.5256/f1000research.157510.r216143

**Published:** 2023-11-20

**Authors:** Francesco Carrer

**Affiliations:** 1 Newcastle University, Newcastle upon Tyne, England, UK

The authors have thoroughly addressed all my comments. The description of the methodology and the conceptual framework underpinning this interesting and very useful piece of software is detailed and clear. I particularly appreciated the technical information about the functionality of Inglamnlagare with regards to CPU and memory allocation, which can be very relevant for end users. I approve the indexing of this paper and I recommend the dissemination of this software.

Are the conclusions about the tool and its performance adequately supported by the findings presented in the article?

Yes

Is the rationale for developing the new software tool clearly explained?

Yes

Is the description of the software tool technically sound?

Partly

Are sufficient details of the code, methods and analysis (if applicable) provided to allow replication of the software development and its use by others?

No

Is sufficient information provided to allow interpretation of the expected output datasets and any results generated using the tool?

Yes

Reviewer Expertise:

Spatial Analysis, Digital Archaeology

I confirm that I have read this submission and believe that I have an appropriate level of expertise to confirm that it is of an acceptable scientific standard.

# Reviewer report: Reviewer response for version 1

**DOI:** 10.5256/f1000research.138901.r178708

**Published:** 2023-08-07

**Authors:** Francesco Carrer

**Affiliations:** 1 Newcastle University, Newcastle upon Tyne, England, UK

The new analytical tool described in this paper is of great relevance for data science in archaeology, as it provides a quick and accurate method to extract information from complex sets of HER data. Although the software is specifically designed for the Swedish HER, its applicability to GPKG formats will extend its use to other digital archives. 

 On the other hand, I believe that more technical details should be included in the description of the software and the dataset it was designed for. For example, the organisation of the entries in the Swedish HER might require additional explanation, possibly supported by figure(s) describing the information associated to each entry. This will facilitate the comprehension of the software application.

 The description of the tool is quite synthetic, and it might be useful to provide some details about the algorithm and its implementation in Python, before moving to the case study. 

 The application of the tool and the results produced are well explained and described, but as I mentioned before, too much space is given to the application of the software compared to its description. Under "Methods", more space is given to the preparation of the datasets in QGIS than to the description of scope and structure of Ing.py.

 The conclusions show that the software has great potential, but more technical information is required to foster its use beyond the Swedish HER.

Are the conclusions about the tool and its performance adequately supported by the findings presented in the article?

Yes

Is the rationale for developing the new software tool clearly explained?

Yes

Is the description of the software tool technically sound?

Partly

Are sufficient details of the code, methods and analysis (if applicable) provided to allow replication of the software development and its use by others?

No

Is sufficient information provided to allow interpretation of the expected output datasets and any results generated using the tool?

Yes

Reviewer Expertise:

Spatial Analysis, Digital Archaeology

I confirm that I have read this submission and believe that I have an appropriate level of expertise to confirm that it is of an acceptable scientific standard, however I have significant reservations, as outlined above.

# Response

**Published:** 2023-10-13

**Authors:** Daniel Löwenborg

**Affiliations:** Uppsala University, Sweden

The authors wish to thank the reviewer for valuable comments and suggestions. We have added an outlook at the end to discuss how the specific case could serve as a more general example. The structure of the software in Python has also been described in more detail in the Methods section. We have contacted the NHB to ask about a technical description of the Swedish HER, but they have no publicly available description that we can use, only a submitted article, in Swedish only. There is some information about HER available from the links in the introduction, but unfortunately, this is also mostly in Swedish.

# Reviewer report: Reviewer response for version 1

**DOI:** 10.5256/f1000research.138901.r156392

**Published:** 2023-03-13

**Authors:** Daniella Vos

**Affiliations:** 1 Department of Cultural Geography, Faculty of Spatial Sciences, University of Groningen, Groningen, The Netherlands

This is a very useful and practical example of how Inglämnlagare can be used to improve the use of an openly accessible dataset in Sweden. However, this is also a very specific case study, and the potential application of this software and approach in other contexts is not addressed (which is OK if this was the original intension, but it might be good to be explicit about the scope of the paper and the use of this software).

 The title of this article sounds like an introduction to Inglämnlagare. However, I see the focus of this paper more as an example for the application of this tool. In any case, it would be good to include a short description of the software, simply referring to another article describing the tool seems lacking in an article devoted to the use of Inglämnlagare. It might also be a good idea to adjust the title to better match the content of this paper. Being explicit about the aim of this paper, and about the intended use of the tool / methodology, can help the reader understand this study.

 Certain statements and sentences are a bit unclear, it might be worthwhile to carefully re-read everything to ensure that there are no vague descriptions or unclear formulations.

 For example, the introduction includes the following statement: "However, access to data is not enough, and it is necessary that data structures are appropriate for the analytical tools used".

 This sentence is a bit unclear. Why is access to data not sufficient? In which context, why? Perhaps you could explain more and relate this statement better to the information that will follow (being explicit about the main aim of this paper / approach would also help tie everything together).

 The discussion does not elaborate a lot about the software and methodology / approach here, which in a way makes sense as this is quite evident and the benefits are pretty straightforward. However, it could have included some information about the choices made throughout the process, alternatives to these, placing this method in a broader context, or addressing the durability (how long do you expect this tool and the site offering the data to remain the same / updated?) and the applicability of the software to other cases.

Are the conclusions about the tool and its performance adequately supported by the findings presented in the article?

Yes

Is the rationale for developing the new software tool clearly explained?

Yes

Is the description of the software tool technically sound?

Partly

Are sufficient details of the code, methods and analysis (if applicable) provided to allow replication of the software development and its use by others?

Yes

Is sufficient information provided to allow interpretation of the expected output datasets and any results generated using the tool?

Yes

Reviewer Expertise:

Digital archaeology

I confirm that I have read this submission and believe that I have an appropriate level of expertise to confirm that it is of an acceptable scientific standard.

# Response

**Published:** 2023-10-13

**Authors:** Daniel Löwenborg

**Affiliations:** Uppsala University, Sweden

The authors wish to thank the reviewer for valuable comments and suggestions. This is indeed a very specific case study, and to make this clearer we suggest to change the title to include the name of the specific database, HER. We have added an outlook at the end to discuss how the specific case could serve as a more general example. The software has also been described in more detail in the Methods section. Note that this is the only article describing the tool and we are not referring to any other article describing the software, only linking to the software itself and the data used.
